# Development of a Resilience Parameter for 3D-Printable Shape Memory Polymer Blends

**DOI:** 10.3390/ma16175906

**Published:** 2023-08-29

**Authors:** Truman J. Cavender-Word, David A. Roberson

**Affiliations:** 1 Polymer Extrusion Lab, The University of Texas at El Paso, El Paso, TX 79968, USA; tjword@miners.utep.edu; 2 Department of Metallurgical, Materials and Biomedical Engineering, The University of Texas at El Paso, El Paso, TX 79968, USA

**Keywords:** shape memory polymers, additive manufacturing, fused filament fabrication, injection molding, self-healing polymers

## Abstract

The goal of this paper was to establish a metric, which we refer to as the resilience parameter, to evaluate the ability of a material to retain tensile strength after damage recovery for shape memory polymer (SMP) systems. In this work, three SMP blends created for the additive manufacturing process of fused filament fabrication (FFF) were characterized. The three polymer systems examined in this study were 50/50 by weight binary blends of the following constituents: (1) polylactic acid (PLA) and maleated styrene-ethylene-butylene-styrene (SEBS-g-MA); (2) acrylonitrile butadiene styrene (ABS) and SEBS-g-MA); and (3) PLA and thermoplastic polyurethane (TPU). The blends were melt compounded and specimens were fabricated by way of FFF and injection molding (IM). The effect of shape memory recovery from varying amounts of initial tensile deformation on the mechanical properties of each blend, in both additively manufactured and injection molded forms, was characterized in terms of the change in tensile strength vs. the amount of deformation the specimens recovered from. The findings of this research indicated a sensitivity to manufacturing method for the PLA/TPU blend, which showed an increase in strength with increasing deformation recovery for the injection molded samples, which indicates this blend had excellent resilience. The ABS/SEBS blend showed no change in strength with the amount of deformation recovery, indicating that this blend had good resilience. The PLA/SEBS showed a decrease in strength with an increasing amount of initial deformation, indicating that this blend had poor resilience. The premise behind the development of this parameter is to promote and aid the notion that increased use of shape memory and self-healing polymers could be a strategy for mitigating plastic waste in the environment.

## 1. Introduction

Humankind has an unparalleled ability to fabricate objects with a wide variety of polymers and manufacturing methods. Components fabricated from polymers are expected to reliably perform in a wide variety of theatres, ranging from components used in aerospace systems to those that support human life in healthcare situations to applications that are considered disposable, such as food packaging and trash bags. However, there are several downsides to society’s use of plastics. A key detractor of the widespread use of plastics is due in part to the way they behave in the environment, particularly in oceans and waterways. The specific gravity of most polymers is close to a value of 1, the same as water, meaning that polymer waste tends to float in water at various depths, depending on the size of the plastic object [1]. Mechanical degradation causes plastic material to break down into smaller and smaller particles, until they reach the microscale. These “microplastics” are ingested by fish and other wildlife and can lead to their death [2]. Chemicals such as phthalate that are commonly found in plastics disrupt the endocrine systems of fish and wildlife and negatively affect their reproductive systems, leading to a negative impact on the population of these animals [3]. The impact of microplastics is not limited to fish and other wildlife, as it is estimated that the amount of microplastics ingested by consumers of seafood is on the order of 11,000 particles per year per person [1]. This indirect consumption of endocrine-harming chemicals by humans can pose a health risk, as plasticizers have been linked to a wide range of endocrine-related health problems and have also been shown to negatively affect fetal development in humans [4]. While in the body, microplastics can further degrade to the nanoscale and be absorbed by body tissue, leading to an immunoreaction [5]. Microplastics have now been found in every ecosystem, meaning that risk of human consumption is not limited to seafood eaters [6]. Even more disturbing is the fact that microplastics have been detected in the fecal matter of humans in several studies [5,6,7,8] and have been linked with inflammatory bowel disease in humans [7]. Perhaps the most shocking discovery related to the impact of polymeric materials on humans is the detection of microplastics in the blood and tissue of human placentas [9], as well as in human whole blood tested from samples obtained from random blood donors [10]. Furthermore, microplastics have been found in the lung tissue of living human beings [11], meaning that plastic waste is literally in the air we breathe! How we handle plastic waste is a big problem. Today plastic refuse can be found on every surface of our world [12,13,14,15]. The “great garbage patches”—essentially floating columns of plastic the size of the state of Texas [16,17,18] in every geographical ocean—are glaring reminders of the negative impact a material type most people use (and discard) every day can have on the environment. Furthermore, a layer of plastic waste is a key characteristic of what is referred to as the “Anthropocene” geological period in which humanity now resides [19]. The ease with which polymeric waste is discarded can easily be illustrated by a ball point pen; when a small amount of ink is used up, a relatively large amount of polymeric material is discarded.

Though recycling is one option for the reduction of polymeric waste, challenges such as contamination by filler materials or mixed plastics; robustness of recycled materials (as compared to virgin material); and the amount of energy needed to sort, separate, and reprocess plastics inhibit the viability of this path [20]. It has recently been reported that only 20% of polymeric material waste is recycled and that the strategy of chemical upcycling of polymeric waste into new materials is a more viable and value-added path [21].

**Figure 1:**
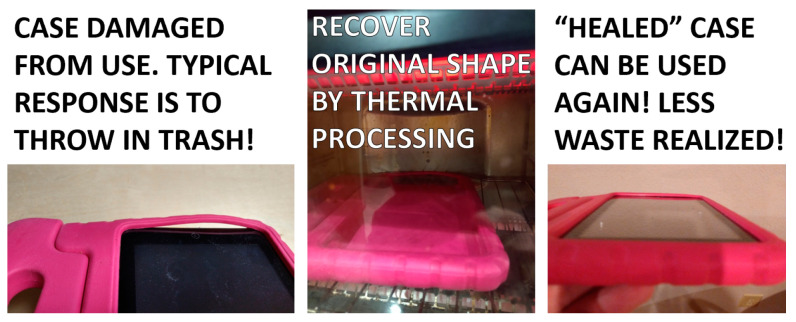
Using the inherent shape memory properties of EVA to enable the reuse of a damaged tablet case and reduce the amount of waste that would have been generated by throwing it away.

The research presented in this work is based on the belief that polymer waste can be reduced by the implementation of polymeric materials with shape memory and self-healing properties, which would allow components fabricated from these plastics to be easily repaired rather than be thrown away. The initial inspiration for this research effort came from a personal experience by one of the authors, involving the protective case of his child’s Amazon Kindle tablet. The case had become mechanically stretched out from use (Figure 1) and the ability to sustain an impact was compromised. The initial thought was to throw the case away and purchase a new one. However, it was found that the case was manufactured from ethylene-vinyl acetate (EVA) foam, a material with shape memory properties and a recovery temperature range between 60 °C and 78 °C [22]. The case was thermally recovered by placing the case in a toaster oven, and the case was then able to be used again. This simple example demonstrates three key aspects related to how society views polymeric materials: (1) the need to overcome what has become an instinctive response related to throwing plastic items away; (2) the need to exploit inherent properties of polymeric materials that may increase the lifespan of components and reduce the frequency of replacement; and (3) the need to proliferate knowledge related to the ability of materials to be easily healed, allowing for plastic part reuse.

Shape memory polymers have been well studied in the literature [23,24,25,26,27,28,29], where the critical parameters used to evaluate the characteristics of a given material system are shape recovery ratio (*R_r_*) and shape fixation ration (*R_f_*), given by the following equations:

(1) $$R_{r}\left(\%\right)=\frac{\varepsilon_{m}-\varepsilon_{p}}{\varepsilon_{m}}\times 100\%$$

(2) $$R_{f}\left(\%\right)=\frac{\varepsilon_{u}}{\varepsilon_{m}}\times 100\%$$

where *ε_m_* is the maximum strain the specimen is subjected to (usually 100% elongation), *ε_u_* is the elongation of the specimen after the load is removed, and *ε_p_* is the elongation of the specimen after the recovery process. To add an aspect of resiliency to the evaluation of shape memory properties, Equations (1) and (2) can be rewritten to include the variable *N*, to include the number of cycles a specimen is subjected to [30]:

(3) $$R_{r}\left(\%\right)=\frac{\varepsilon_{m}-\varepsilon_{p}\left(N\right)}{\varepsilon_{m}}\times 100\%$$

(4) $$R_{f}\left(\%\right)=\frac{\varepsilon_{u\left(N\right)}}{\varepsilon_{m}}\times 100\%$$

The characterization of self-healing of polymers can be made by calculating the self-healing efficiency, which is calculated using various methodologies but generally involves the comparison of mechanical properties of specimens that have been severed into two pieces and then healed with the mechanical properties of control specimens [31]. For example, a work conducted by Xu et al. [32] involving a shape memory polymer blend system of thermoplastic urethane (TPU) and polycaprolactone (PCL) with various amounts multi-wall carbon nanotubes (MWCNTs) determined the self-healing efficiency by comparing the Young’s modulus of control specimens with those that were healed after cutting and following a facile equation and a methodology as described by Wool and O’Connor [33]:

(5) $$R\left(E\right)=\frac{E_{cut}}{E_{pristine}}\times 100\%$$

where *R*(*E*) is the self-healing efficiency based on the Young’s modulus. Following the same concept, percent elongation (%El) and ultimate tensile strength (UTS) can also be used to determine the self-healing efficiency of a polymer using the following equations:

(6) $$R\left(\varepsilon \right)=\frac{\varepsilon_{cut}}{\varepsilon_{pristine}}\times 100\%$$

(7) $$R\left(\sigma \right)=\frac{\sigma_{cut}}{\sigma_{pristine}}\times 100\%$$

where *ε* and *σ* refer to the %El at break and UTS, respectively [33,34]. Expressing in terms of a percent gives a quantifiable metric for determining the ability of a polymeric material to retain a given physical property, *E*, *σ*, *ε*, among others, after being subjected to a healing process. However, considering the example mentioned above related to the tablet case, damage to a polymer great enough to cause a component to be discarded as waste does not necessitate total breakage, rather deformation of a polymer component can render it useless. Therefore, developing a parameter based on how well a material can be recovered from various levels of damage is essential for understanding the resilience of a polymeric material with shape memory and self-healing properties.

Interest in the use of 3D printable SMP blends is increasing, with recent examples in the literature utilizing similar material systems as those used in the work presented here. For example, Rahmatabadi et al. [35] explored the effect of varying the temperature at which the permanent shape was programmed on the shape memory properties of a polylactic acid (PLA)/(TPU) blend where the components were manufactured by FFF. Additionally, the effect of environmental conditions on shape memory properties is also gaining interest. For example, Garces et al. [36] explored the effect of moisture exposure on the shape memory properties of FFF-manufactured TPU specimens. A work by Avila et al. [37] explored the effect of moisture exposure at an elevated temperature on the shape memory properties of a blend composed of PLA and styrene ethylene butylene styrene with a maleic anhydride graft (SEBS-g-MA) and found that there was no degradation to the shape memory properties. Another work by Rahmatabadi et al. [38] explored the relationship between the number of shape memory cycles and shape memory properties for composite structures composed of either acrylonitrile butadiene styrene (ABS) and TPU or PCL and TPU, and they found that the shape memory properties deteriorated as the number of cycles increased

The work presented here demonstrates the development of a resilience parameter that can be used to quantify the ability of a material to retain mechanical properties after thermal recovery from damage. This work is part of a larger effort presented in [39]. Here, SMP blends were explored: (1) a SMP blend composed of ABS SEBS-g-MA in a 50:50 by weight ratio, whose shape memory properties were originally characterized by Andrade Chávez et al. [29]; (2) a SMP blend composed of PLA and TPU in a 50:50 by weight ratio, originally characterized by Quiñonez et al. [23]; and (3) a SMP blend composed of PLA and SEBS-g-MA, also first demonstrated by Quiñonez et al. [23]. We chose to use these three blends because we have already characterized in great detail the shape memory properties and the effect of FFF print raster patterns on these properties [23,29]. In this work, the resilience parameter refers to the ability of this material to recover from different levels of damage and retain mechanical properties, where the property we will focus on in this study is the ultimate tensile strength (UTS). The effect of the manufacturing process on material resilience was also explored by comparing specimens that were fabricated through the additive manufacturing (AM) process of fused filament fabrication (FFF) with those that were manufactured through injection molding (IM).

The motivation for the development of this resilience parameter is to provide a tool to evaluate the ability of a polymeric material to retain its mechanical properties after being recovered from damage. We believe this parameter can be applied to materials beyond those examined here. We believe that increased use of repairable plastics will enable a paradigm shift in the way society views the use of polymeric materials, from cheap and disposable to resilient and reusable.

## 2. Materials and Methods

Though we have already characterized the shape memory and mechanical properties for the blends explored here in previous works [23,29], we have included the shape memory properties, as the previous works only based the shape memory characterization on samples that were subjected to a strain of 100% elongation. Additionally, the previous works subjected the specimens to a 5 min dwell time. Here, we explore the effect of % elongation amount prior to recovery, as well as not keeping the specimens under a static load, on the tensile and shape memory properties. The key variables that influenced the mechanical and shape memory properties and the resilience parameter developed here are summarized in Table 1.

### 2.1. Filament Fabrication

The PLA used in this study was acquired from NatureWorks, LLC (Ingeo Biopolymer Grade 4043D, NatureWorks, LLC, Minnetonka, MN, USA) in the form of pellets. The specific grade, 4043D, was chosen because it is regarded as a commercially pure form of PLA, free of additives such as crystallization promoters or impact modifiers, in addition to being the grade commonly used in the FFF process [40]. The SEBS-g-MA (hereinafter referred to as SEBS) used in this study was Grade FG1901-GT, (Kraton, Houston, TX, USA) in pellet form. The TPU used in this work was off-the-shelf FFF filament supplied by NinjaFlex (Fenner, Inc., Manheim, PA, USA). The ABS used in this work was supplied by SABIC (grade MG94, SABIC, Pittsfield, MA, USA) in the form of pellets. This grade was chosen because it is the grade commonly used by FFF filament manufacturers More details pertaining to the ABS/SEBS blend can be found elsewhere in the literature [29,41]. Additionally, we have demonstrated the use of various forms of SEBS as a suitable modifier of rigid polymers in the development of FFF-specific feedstock materials [25,29,41,42,43,44,45].

To facilitate melt compounding, the TPU in filament form was first pelletized in ambient conditions using a Collin Teachline strand pelletizer (Collin Lab and Pilot Solutions, Norcross, GA, USA). Prior to melt compounding and filament extrusion, all four materials were dried in a compressed air dryer (Dri-Air CFAM Micro-Dryer, East Windsor, CT, USA) using a schedule we had used previously, where PLA was dried for 2 h at 50 °C, ABS was dried for 4 h at 80 °C, SEBS-g-MA was dried for 4 h at 60 °C, and TPU was dried for 4 h at 60 °C. Once fully dry, three different polymer blends were created using a Collin twin screw extruder/compounder (model ZK-25T): a 50:50 by weight blend of PLA and SEBS, a 50:50 by weight blend of ABS and SEBS, and a 50:50 by weight blend of PLA and TPU. Each of the blends was extruded to a target diameter of 2.85 mm for use in our FFF-type 3D printers. The extrusion parameters for each blend are shown in Table 2 below.

### 2.2. Specimen Fabrication

**Figure 2:**
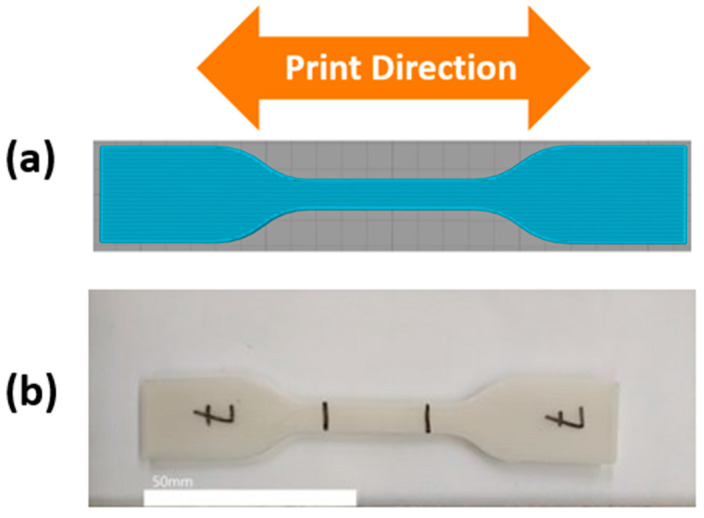
(**a**) Print raster pattern for specimens that were manufactured using FFF in this study and (**b**) example of a FFF-made specimen.

Tensile test samples were fabricated according to ASTM D638 Type IV [46] geometry using two different methods: FFF-type 3D printing and traditional injection molding. Samples were additively manufactured with a Lulzbot Taz 5 3D printer (Fargo Additive Manufacturing Equipment 3D, LLC, Fargo, ND, USA) equipped with a single extruder with a 0.5 mm diameter nozzle. Injection molded specimens were fabricated using a manual piston-type injection molding machine (Model 150A, LNS Technologies, Scotts Valley, CA, USA) and a custom machined ASTM D638 Type IV aluminum mold. Samples for analysis via DMA were additively manufactured according to the ASTM D4065 [47] standard. Both the additively manufactured tensile specimens and the DMA specimens were fabricated with a longitudinal raster pattern, where the print rasters were parallel with the length of the specimen (Figure 2). We chose this raster pattern because we have found that the FFF process aligns the polymer phases parallel to the raster direction and that the longitudinal raster pattern yields the most robust strength and shape memory properties [23,29]. All additively manufactured tensile samples and DMA specimens were printed with 100% infill. We chose the longitudinal raster pattern in particular for the tensile specimens because the direction of applied force during the tensile test acts parallel to the raster direction, and we have found this raster pattern to provide optimal results compared to other print raster schemes [23,29,48]. The printing parameters for each material blend are shown in Table 3. We established these parameters during the initial development of these blends [23,29,41]. The temperatures at which each material was injection molded and at which the aluminum mold was preheated are shown in Table 4.

### 2.3. Mechanical Testing

DMA testing was performed using a PerkinElmer DMA 8000 (PerkinElmer, Waltham, MA, USA) using a dual cantilever setup following ASTM D4065 operating parameters and a temperature sweep appropriate for each material blend. The temperature range selected for testing needed to encompass the glass transition temperatures of both polymers in each given blend, which was below 0 °C for every elastomeric component. The low temperature was achieved using a liquid nitrogen dewar connected to the testing apparatus and controlled by the DMA software (Pyris version 10.1.0.412). The DMA data were used to determine the temperature used for the shape memory recovery process. The DMA test parameters are shown in Table 5.

Tensile data for each material system were collected according to the ASTM D638 standard using an MTS Criterion C-44 tensile testing machine equipped with a 10 kN load cell and an Advantage™ Model AHX 800 extensometer (MTS Systems Corporation, Eden Prairie, MN, USA). Baseline tensile data were collected for each of the three material blends in both additively manufactured and injection molded forms. The sample pool size for the initial tensile testing and shape memory characterization was five specimens. For experiments involving a dwell time, the specimen pool sample amount was reduced to 3 specimens, due to material availability constraints. To evaluate the effect of the shape memory recovery process on the tensile strength of each polymer blend, three batches of samples were deformed to predetermined percentages of their gauge lengths (25%, 50%, and 100%) and then pulled to failure after they had been recovered at elevated temperature in an oven. The results of these tensile tests were compiled and a trendline was created to fit the change in tensile strength as a function of the amount of deformation from which the samples recovered. Further details of this process will be explained later.

### 2.4. Shape Memory Characterization

**Figure 3:**
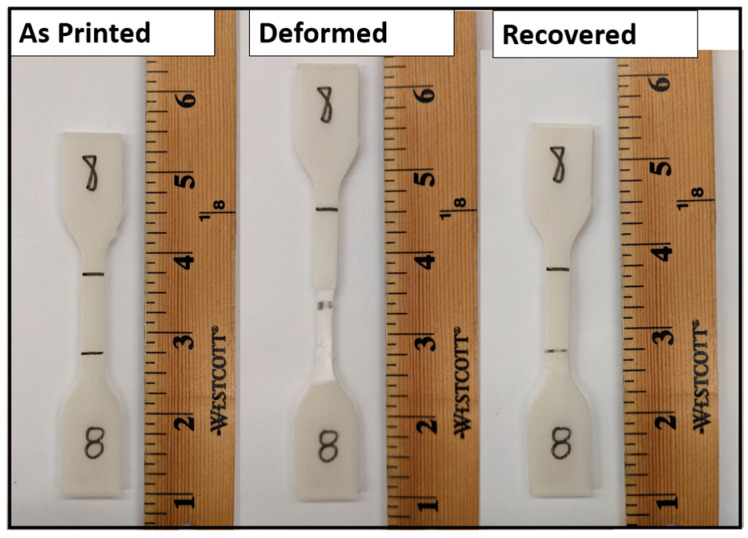
Example of the shape memory cycle.

Shape memory properties were evaluated using methods previously established in previous works conducted by our group [23,29,37]. Three batches of different elongation percentages were pulled in the tensile tester to different amounts of elongation: one batch was pulled to 100% of the specified 25 mm gauge length, the second batch was pulled to 50% of the gauge length, and the final batch was pulled to 25% of the gauge length. To ensure each specimen received the necessary amount of strain, the tensile tester was programmed to stop at the necessary displacement for each of the desired elongation percentages. Two separate studies were performed to elucidate the effect of dwell time at maximum strain: (1) a group of samples that were promptly removed from the tensile tester once the intended amount of elongation was reached; and (2) a group of samples that was allowed to dwell at maximum strain for 5 min. Recovery of the original shape was achieved by placing the specimens in a horizontal air flow oven (Model 3.65, VWR International, Radnor, PA, USA) at temperatures determined according to the results of the DMA testing for each material blend. After recovery, the samples were then pulled to failure in the tensile tester. Length measurements were taken before deformation, after deformation, and after recovery for shape memory property calculation purposes using a precision digital caliper. Shape memory properties were analyzed by calculating *R_f_* (Equation (1)) and *R_r_* (Equation (2)), as described previously. A high-level depiction of the shape memory cycle is shown in Figure 3.

### 2.5. SEM Microanalysis

After mechanical testing, the fracture surfaces of representative samples were analyzed through scanning electron microscopy (SEM) using a Hitachi SU-3500 Variable Pressure SEM (Hitachi America, Ltd., Santa Clara, CA, USA). Due to charging of the samples, imaging was performed at a pressure of 90 Pa either using a backscattered electron detector (BSE) or an ultra-variable detector (UVD) at an accelerating voltage between 10 and 15 kV.

## 3. Results

### 3.1. Dynamic Mechanical Analysis

**Figure 4:**
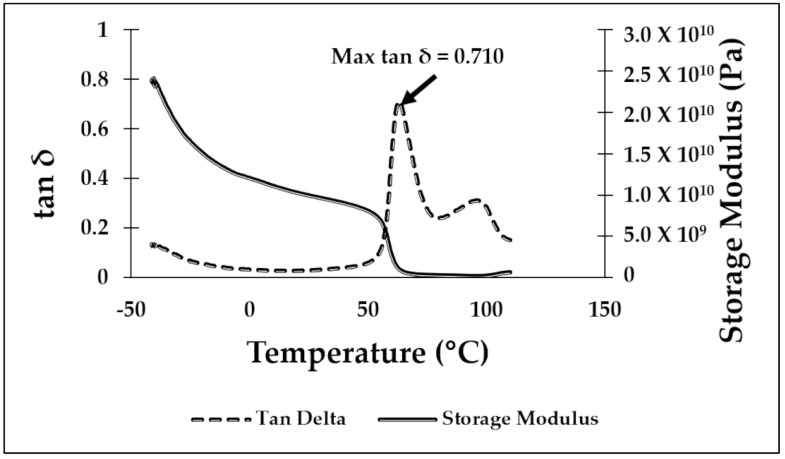
DMA graph for the 50/50 PLA/SEBS blend.

**Figure 5:**
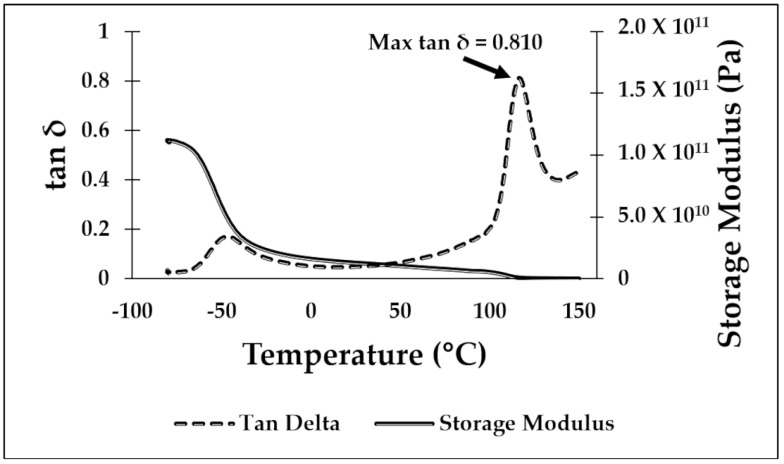
DMA graph for the 50/50 ABS/SEBS blend.

**Figure 6:**
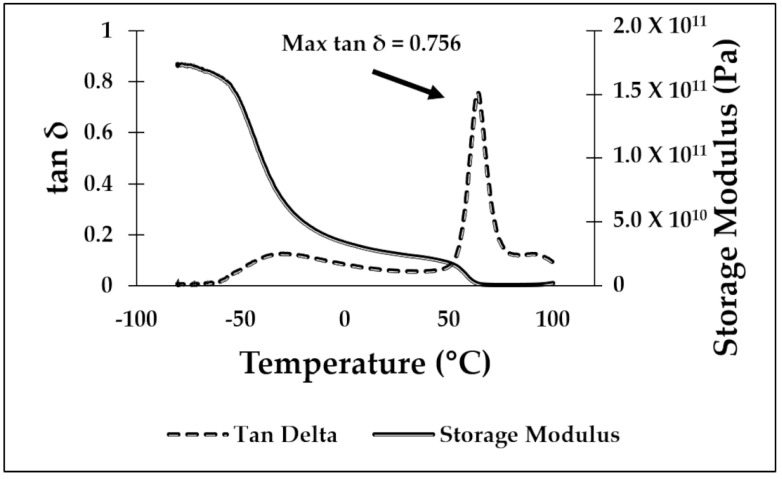
DMA graph for the 50/50 PLA/TPU blend.

DMA testing was used to determine the temperature for the shape recovery processing. Values obtained during testing were the max tan δ temperature and the glassy onset temperature, as determined from the storage modulus drop off as described in the ASTM D4065 standard [49]. In previous work performed by our group [23,29], the glassy onset temperature was considered the best temperature to deform a specimen to a temporary shape and the temperature at which the max tan δ is reached to be the recovery temperature used to return a specimen to the original, or “programmed”, shape for shape memory characterization. However, in this study, we focused on room temperature deformation, so the critical temperatures were those at which the max tan δ occurred. The DMA curves for PLA/SEBS, ABS/SEBS, and PLA/TPU are shown in Figure 4, Figure 5 and Figure 6, respectively. As shown in previous studies, exposing the sample to a temperature above that of the max tan δ will induce the shape recovery process. The results gathered from the DMA testing showed a max tan δ value of 0.710 at 63.08 °C for the PLA/SEBS blend, a max tan δ value of 0.810 at 116.23 °C for the ABS/SEBS blend, and a max tan δ value of 0.756 at 63.88 °C for the PLA/TPU blend. As seen in Figure 4, there are two peaks in the tan delta curve for the PLA/SEBS blend, which likely indicates that the two polymers did not mix well. Previous work has shown that PLA and SEBS do not mix well, as was documented with scanning transmission electron microscopy (STEM) imaging, which showed that there were multiple phases that were not well dispersed in a microstructure that resembled oil mixed with water [23]. A recovery temperature of 70 °C was selected for both the PLA/SEBS and PLA/TPU blends, while a recovery temperature of 120 °C was selected for the ABS/SEBS system, as these temperatures were slightly above the temperatures at which the max tan δ occurred.

### 3.2. Mechanical Testing

#### 3.2.1. PLA/SEBS

*Additively Manufactured:* The PLA/SEBS blend showed the highest average tensile strength in the as-printed form, with an average of 25.26 ± 0.27 MPa. The average tensile strength decreased with the increasing amount of deformation from which the sample recovered. After recovering from 25%, 50%, and 100% elongation, the average tensile strength dropped to 24.68 ± 1.19, 23.38 ± 2.31, and 21.98 ± 0.61 MPa, respectively, for the samples with no dwell time. The dwell sample pool showed similar results, with the average tensile strength decreasing from 23.43 ± 1.27, to 24.26 ± 4.61, to 21.1 ± 0.54 MPa for 25%, 50%, and 100% elongation, respectively. Keeping the specimens under load at a given percent elongation did not make a significant difference in the tensile strength exhibited after a thermal recovery cycle.

**Figure 7:**
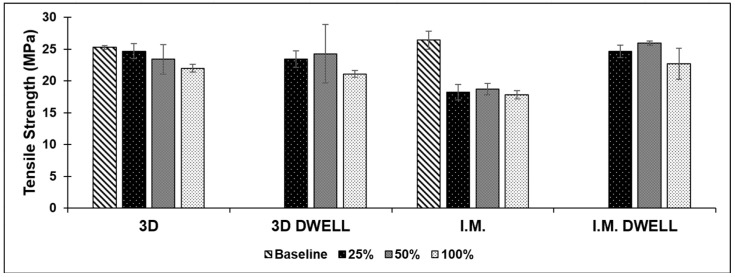
Comparison of average tensile strength for all test batches of the PLA/SEBS blend.

*Injection Molded:* Similarly to the additively manufactured samples, the injection molded samples showed their highest average tensile strength in the as-fabricated form, with consistently decreasing values as the amount of deformation from which they recovered from increased. The as-fabricated specimens had a tensile strength of 26.4 ± 1.4 MPa. The rate of decrease in tensile strength with the increase in deformation amount also mirrored those shown in the 3D printed samples, with the tensile strength dropping from 24.6 ± 0.95, to 25.9 ± 0.33, to 22.67 ± 2.46 MPa for 25%, 50%, and 100% elongation, respectively. Dwell time did not make a significant difference for the tensile strength, which decreased as the amount of strain increased. The batch of samples that were not allowed to dwell showed an overall lower tensile strength compared to the dwell sample pool. The results of the tensile testing for this blend are shown in Figure 7.

#### 3.2.2. ABS/SEBS

*Additively Manufactured:* The ABS/SEBS blend showed very consistent tensile strength in its 3D printed form, regardless of the amount of deformation from which the samples were recovered. The tensile strength was 20.42 ± 0.41, 20.56 ± 0.22, and 20.3 ± 1.48 MPa for the 25%, 50%, and 100% deformation batches, respectively, when the samples were not allowed any dwell time. Considering a baseline tensile strength of 20.32 ± 0.62 MPa, the blend showed an impressive ability to maintain its strength, even after deformation and recovery. Interestingly, adding a five-minute dwell time under load at the prescribed amount of deformation led to a very small increase in strength as the amount of deformation increased. The average tensile strength for the samples in this group was 19.23 ± 0.26, 19.43 ± 0.12, and 20 ± 0.59 MPa for the 25%, 50%, and 100% deformation groups, respectively. Though the strength increased along with the amount of deformation recovery, the samples never quite reached their baseline tensile strength, indicating that the addition of a dwell time may have very slightly weakened the samples.

**Figure 8:**
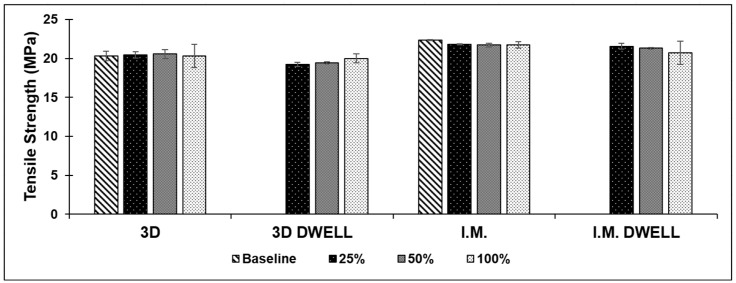
Comparison of average tensile strength for all test batches of the ABS/SEBS blend.

*Injection Molded:* The injection molded batches of samples, both with and without dwell time in the test schedule, showed a very consistent average tensile strength, as the amount of deformation from which they were recovered increased. The average tensile strength for the samples without dwell time was just slightly lower than that of the baseline tensile strength exhibited by the as-fabricated samples: 22.32 ± 0.07 MPa. The batch of samples that were not allowed to dwell showed an average tensile strength of 21.82 ± 0.07, 21.7 ± 0.24, and 21.74 ± 0.40 MPa for the 25%, 50%, and 100% deformation groups, respectively. The batch of samples which were allowed to dwell for five minutes at load showed an average tensile slightly lower than that of both the baseline and no-dwell samples. The average tensile strength was 21.56 ± 0.40, 21.3 ± 0.08, and 20.73 ± 1.51 MPa for the 25%, 50%, and 100% deformation groups, respectively. Considering error, the ABS/SEBS blend did not exhibit a relationship between the amount of strain prior to recovery and tensile strength, retaining its mechanical properties. The graphical results for the tensile testing of the ABS/SEBS blend are seen in Figure 8.

#### 3.2.3. PLA/TPU

**Figure 9:**
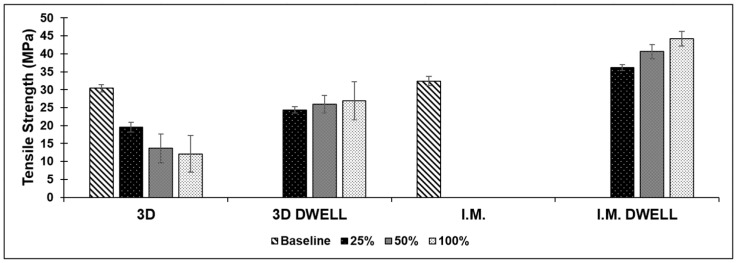
Comparison of average tensile strength for all test batches of the PLA/TPU blend.

*Additively Manufactured:* Tensile testing of the PLA/TPU blend yielded inconsistent mechanical properties when comparing sample pools based on the amount of pre-recovery strain, as well as comparing the two fabrication methods. The average tensile strength for this blend in the as-printed form was 30.42 ± 1.03 MPa. Subjecting the samples to the deformation and recovery process led to a very significant decrease in average tensile strength. In the case of 3D printed specimens, however, the dwell sample pool exhibited a much higher average tensile strength compared to the non-dwell sample pool. The dwell sample pool also exhibited an increase in strength with the increase in dwell time, but the difference was not statistically significant. As shown in Figure 9, there was a trend indicating that the strength decreased when not allowed to dwell and increased when allowed to dwell. The no-dwell sample pool showed an average tensile strength of 19.52 ± 1.38, 13.64 ± 3.96, and 12.12 ± 5.15 MPa for the 25%, 50%, and 100% deformation groups, respectively, while the samples in the dwell sample pool had an average tensile strength of 24.36 ± 0.90, 25.96 ± 2.46, and 26.9 ± 5.33 MPa for the 25%, 50%, and 100% deformation groups, respectively. Though the difference was not statistically significant, there was a trend indicating an increase in overall tensile strength with the addition of dwell time to the test schedule.

*Injection Molded:* Tensile testing of injection molded specimens also yielded inconsistent results. The first batch of blended material was used in the non-dwell sample pool. The samples that were injection molded ruptured well before reaching their prescribed amount of deformation. We believe this was the result of hygroscopicity, as both PLA and TPU are extremely sensitive to this phenomena, which would lead to a loss of mechanical properties [50,51], and there was a large amount of time between material compounding and material testing, due to the global pandemic. The material that was used in the dwell experiments was tested almost immediately after compounding, and this batch of samples was able to reach their prescribed amount of deformation without issue and showed a greatly increased average tensile strength, along with a trend of increasing strength with increasing amount of deformation recovery, as compared to the 3D printed counterparts. The injection molded samples allowed to dwell showed an average tensile strength of 36.16 ± 0.87, 40.63 ± 1.96, and 44.26 ± 2.03 MPa for the 25%, 50%, and 100% deformation groups, respectively. The baseline tensile strength for this polymer blend in the as-fabricated condition was 32.42 ± 1.32 MPa, which is less than any of the samples that were subjected to the shape memory recovery test.

A bar graph comparing the average tensile strength of this polymer blend in both fabrication methods at all amounts of deformation is shown in Figure 9.

### 3.3. Shape Memory Property Characterization

#### 3.3.1. PLA/SEBS

The PLA/SEBS blend exhibited the highest fixation ratio of all the blend systems tested in this study, with the highest being in the 3D printed form and subjected to a dwell time of 5 min at the prescribed amount of strain. For the 3D printed samples, the addition of the 5 min dwell time led to a significant increase in fixation ratio, which decreased in magnitude with the increase in deformation amount. The recovery ratio of this blend in 3D printed form showed a negligible change across both fabrication methods, whether or not the samples were allowed to dwell. Most of the samples had a recovery ratio slightly over 100%, indicating a small amount of over-recovery, or shrinkage, occurring during the recovery process.

The sample pool that yielded the lowest values in terms of shape memory properties for this material blend was the injection molded samples from the non-dwell sample pool. Though the *R_r_* was equal or slightly greater than 100%, the average *R_f_* values ranged from 60% to 74.3%. It is notable that the *R_f_* values increased as the amount of deformation prior to thermal recovery increased. The best performing batch of PLA/SEBS samples in the context of shape memory properties was the 3D printed specimens from the dwell sample pool, as applying a dwell time of 5 min increased the fixation ratio as compared to the non-swell sample pool. The average measured shape memory properties for the PLA/SEBS blend are presented in Table 6 (A video illustrating the shape memory properties of the PLA/SEBS blend can be seen at Supplementary Materials).

#### 3.3.2. ABS/SEBS

Beginning with the 3D printed specimen pools, the addition of a dwell time had the effect of increasing the fixation ratio for all specimens. The fixation ratio was nearly identical for the samples which were stretched to 50% of their gauge length and, contrary to the 25% samples, the fixation ratio decreased very slightly when dwell time was added for the samples that were stretched to 100% of their gauge length. There was no difference in the recovery ratio between the sample batches, regardless of dwell time or deformation amount, and all the samples had the tendency to over-recover and end up with a final length just slightly shorter than they were when printed.

The injection molded samples showed a similar trend to the 3D printed samples but with more consistency. The fixation ratio of the samples increased for all batches subjected to dwell time but at a decreasing rate with increasing deformation amount. The fixation ratio increased by 12% for the 25% samples, 8% for the 50% samples, and 5% for the 100% samples. In a similar manner as observed for the PLA/SEBS blend, the recovery ratio for these samples stayed the same regardless of the dwell time or deformation amount, and the values mirrored those of the 3D printed samples. The measured shape memory properties for the ABS/SEBS blend are seen in Table 7.

#### 3.3.3. PLA/TPU

The PLA/TPU polymer blend, much like the PLA/SEBS blend, showed a significant improvement in fixation ratio when a dwell time was added to the testing schedule for the 3D printed samples. This improvement in fixation ratio was very similar in magnitude for all three deformation amounts. The recovery ratio was nearly identical for both batches of 3D printed samples, indicating that the ability of the samples to recover their original shape was not affected by the dwell time.

Every sample in the first batch of injection molded samples, intended to be tested without any dwell time at load, ruptured well before reaching the prescribed amount of strain. This lack of ductility led to an inability to obtain shape memory data for this blend at that time. As mentioned previously, we believe the reason for the inability of this sample pool to sustain strain was due to hygroscopicity. The second batch of injection molded samples, used for testing with a five-minute dwell time at load, showed significantly more ductility and all samples were able to achieve their prescribed amount of strain. These samples showed the best shape memory properties for this blend. The injection molded samples allowed to dwell showed an increase in fixation ratio over both 3D printed sample batches at all amounts of deformation. The recovery ratio also was the same across both fabrication methods, regardless of dwell time. The shape memory properties for the PLA/TPU blend are tabulated in Table 8.

### 3.4. SEM Microanalysis

The results of the SEM fractography were mostly as expected for the polymer blends composed mainly of rubbery materials, with the exception being the first batch of injection molded PLA/TPU samples, which all failed well before the lowest amount of prescribed strain. The SEM microanalysis performed here was a high-level analysis of the fracture surface of the control specimens. For a more comprehensive microstructural and fractographic analysis of the materials studied here, we encourage the reader to refer to references [29,37,41] for the ABS/SEBS system and reference [23] for the PLA/SEBS and PLA/TPU systems.

**Figure 10:**
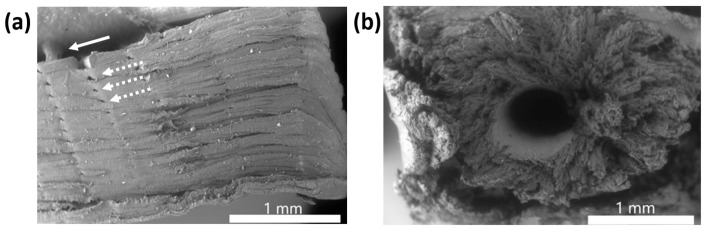
(**a**) Fracture surface of a FFF specimen made from PLA/SEBS where a fibril is indicated by the solid white arrow and print void defects are highlighted by dashed arrows, and (**b**) fracture surface of an injection molded PLA/SEBS specimen.

Beginning with the PLA/SEBS blend manufactured via 3D printing (Figure 10a), the fracture surface looks planar and resembles a brittle failure mode, but the reason for the lack of plastic deformation on the fracture surface was the necking of the specimen which eventually failed due to a reduction in cross-sectional area [41]. The presence of a fibril (highlighted by the solid white arrow in Figure 10a) is a feature associated with ductile failure modes [40,43,51,52]. Print-related void defects (highlighted by dashed arrows in Figure 10a) are also visible on the fracture surface, which manifest between the print raster beads. The interbead voids are very small, indicating that the print parameters were very well optimized as the individual beads fused together well. The injection molded samples of PLA/SEBS exhibited a shrinkage void in the center of the volume of the gauge section (Figure 10b). This void was due to the injection molding process and likely due to the injection molder used being piston-based rather than screw-based [23]. The fracture surface morphology from the edge of the void to the surface of the specimen is dominated by a large amount of plastic deformation and fibrils, which is consistent with ductile failure. The edge of the specimen has a different morphology. The contrast in fracture evidence between the inner and outer volume of the sample shows a common phenomenon of injection molding, where the material nearest the mold wall cools down faster than the material in the center.

**Figure 11:**
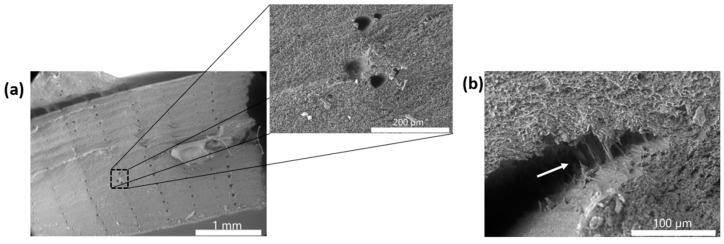
(**a**) Fracture surface of a FFF specimen made from ABS/SEBS, and (**b**) fracture surface of an injection molded ABS/SEBS specimen where fibrils are indicated by the white arrow.

The ABS/SEBS blend showed very consistent fracture surfaces with both manufacturing methods, with a predominance of ductile failure features. The FFF (Figure 11a) exhibits a fracture surface morphology similar to that of the PLA/SEBS blend, where a reduction in surface area yielded a morphology that resembled a brittle fracture surface. There was also the presence of print raster beads. The higher mag inset (Figure 11a) shows the print raster voids in greater detail, as well as the presence of small fibrils. The injection molded specimen (Figure 11b) also exhibited microfibrils. Fibrils also manifested laterally (highlighted by white arrow in Figure 11b) as the specimen began to fail, due to the strain induced by the tensile test.

**Figure 12:**
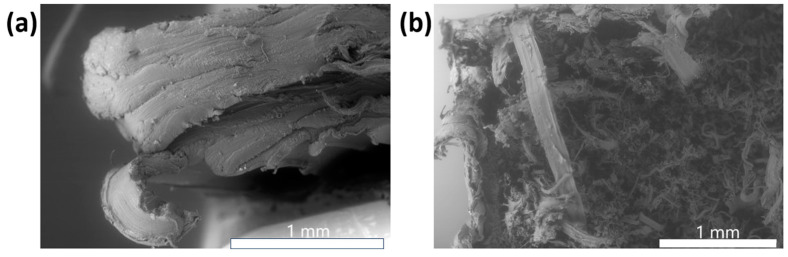
(**a**) Fracture surface of a FFF specimen made from PLA/TPU, and (**b**) fracture surface of an injection molded PLA/TPU specimen.

Finally, the PLA/TPU blend showed very different fracture surface features between the 3D printed and the injection molded samples. The 3D printed samples showed significant delamination between the layers of polymer and a great degree of deformation (Figure 12a). These features are characteristic of a ductile fracture surface, which is to be expected as this blend is 50% thermoplastic polyurethane by weight and this is consistent with other works with this material system [23]. The injection molded specimen (Figure 12b) is dominated by the presence of fibrils, again a feature associated with a ductile failure mode.

### 3.5. Calculation of the Resilience Parameter

In this study, four different polymers were combined in three different blends and fabricated into test specimens using two different methods. Each polymer blend performed uniquely in terms of mechanical properties and shape memory properties. The best performer in terms of raw tensile strength, regardless of whether the samples were deformed and recovered, was the PLA/TPU blend in the injection molded form after recovering from 100% deformation with a five-minute dwell time under load. If we were to rank the material systems in terms of combination of polymers, fabrication method, and dwell time, the second-best performers were all of the PLA/SEBS blends, except the no-dwell injection molded sample pool, as well as the 3D printed PLA/TPU blend from the dwell sample pool. The ABS/SEBS polymer blend had the lowest average tensile strength across all batches, but was also the most consistent, with the lowest value being 19.23 ± 0.26 MPa and the highest being 21.82 ± 0.07 MPa. This blend showed the least sensitivity to recovery from deformation of all the blends.

Possibly the most significant result from this study was the development of a measurable mechanical property we are calling the “resilience parameter”. This parameter is calculated by averaging the tensile strength of each group of samples and plotting those values as points on a graph with the *y*-axis being tensile strength and the *x*-axis being the percent elongation from which the group of samples was recovered. Next, plotting a line of best fit to those points gives a linear equation describing the change in strength vs. percent deformation recovered. The slope of this line being negative indicates a reduction in average tensile strength as the samples are exposed to, and recovered from, greater amounts of deformation. The slope being positive indicates the opposite: an increase in strength as the deformation amount increases. Provided that the line of best fit matches the data set well, by having an R^2^ value close to 1 or 0, the *y*-intercept of the line will be nearly the same as the baseline tensile strength.

**Figure 13:**
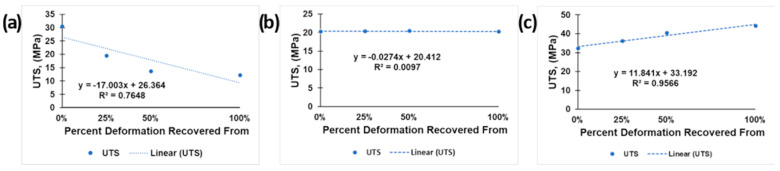
Example of linear curve fitting for materials with (**a**) poor resilience, the 3D printed PLA/TPU from the no-dwell sample pool; (**b**) good resilience, 3D printed ABS/SEBS that was not held at a 5 min dwell time under load; and (**c**) excellent resilience, injection molded PLA/TPU that was held for a 5 min dwell time under load.

To create a unitless value for this parameter, we divide the slope of the line, *m*, by the *y*-intercept, *b*, while assuming the value of *x* to be equal to 1. The result is a value that indicates the percent of baseline tensile strength that is lost when deformed to 100% of the gauge length and recovered for 5 min at the established recovery temperature. This value was calculated for each of the polymer blends for both manufacturing methods, both with and without dwell time. A graphical representation of the calculation of the parameter is presented in Figure 13 for a material/process combinations with poor, resilience (Figure 13a), good resilience (Figure 13b) and excellent resilience (Figure 13c), and the data for all materials and manufacturing combinations tested here are tabularized in Table 9.

This resilience parameter value can be interpreted as a larger number being more favorable. If the resilience parameter is negative, then the material loses strength as the deformation recovery is increased, and the magnitude of that value indicates the rate at which strength is lost. If the resilience parameter is near to or equal to zero then the tensile strength of the material is unaffected by the deformation and recovery process. If the resilience parameter is positive, then the tensile strength of the material increases as the amount of deformation recovered increases. For the purposes of evaluating the ability of a material to maintain its strength after deformation and recovery, a higher number indicates a better performance than a lower number. By comparing these values, a trend appears for each blend. The PLA/SEBS blend performs better when the sample is allowed to dwell, regardless of the manufacturing methods. However, both manufacturing methods showed a negative resilience parameter in all cases, with only minor variation in magnitude, with the highest being −10.69 in the injection molded form without dwell time and the highest being −14.97 in the 3D printed form with dwell time. The ABS/SEBS showed an interesting contrast in performance between manufacturing methods. The 3D printed sample groups had nearly the same resilience parameter of barely below 0 whether they were allowed to dwell or not. While the 3D printed samples showed no loss of strength after deformation and recovery, the injection molded samples showed a consistent decrease in strength, with the no-dwell sample pool faring slightly worse than the dwell sample pool, with values of −10.69 and −6.75, respectively. The PLA/TPU blend was the only sample group with a positive resilience parameter value, as well as the greatest range of resilience parameters between sample groups. The 3D printed sample groups varied greatly between the dwell and no-dwell samples, with values of −7.38 and −64.49, respectively. These values showed a major sensitivity to dwell time in terms of retained tensile strength after deformation recovery for the 3D printed samples. The PLA/TPU blend from the injection molded sample pool that was subjected to a 5 min dwell under load prior to thermal recovery showed the only positive resilience parameter for this study, with a high value of 35.67. This was the only sample group to show an increase in strength with an increased amount of deformation from which the samples were recovered.

## 4. Discussion

**Figure 14:**
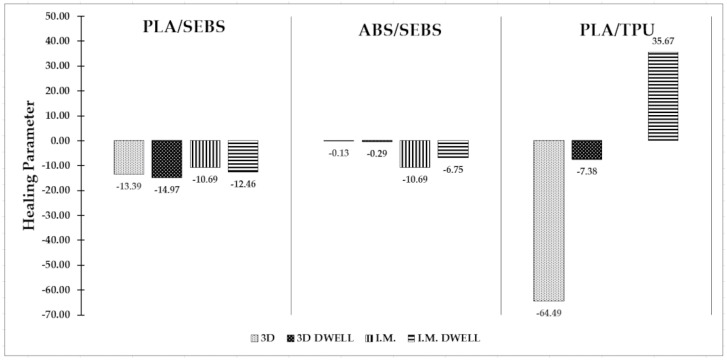
Graphical representation of the resilience parameter for the materials, manufacturing methods, and deformation processes used in this study.

Graphical representation of the resilience parameter for the materials and manufacturing methods tested here (Figure 14) allows the performance trends to be seen very easily. It is easily seen that the PLA/SEBS test batches were the most consistent in performance, with dwell time being the most important variable. The blend of ABS/SEBS showed an easily observable pattern, in which the injection molded samples performed worse than the 3D printed samples. As mentioned previously, we believe the increase in strength observed in the PLA/TPU blend specimens that were injection molded and allowed to dwell under load before being subjected to a recovery process was related to an increase in crystallinity caused by the thermomechanical processing.

**Figure 15:**
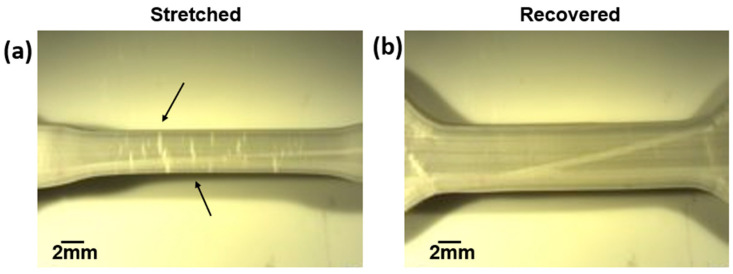
Optical micrograph of the gage section of a PLA/SEBS tensile specimen in (**a**) the stretched condition where the black arrows highlight craze cracking and (**b**) after thermal recovery. Note the disappearance of craze cracks in the gage section after recovery.

The use of the resilience parameter has implications for the measurement of the self-healing ability of polymers where the material has not been severed into two pieces. We have noted elsewhere that the PLA-SEBS blend exhibits an ability to recover from strain whitening caused by craze cracking [37] and an example of this is seen in the optical micrographs in Figure 15. This is an important scenario to consider, especially in the context of waste reduction because, as was pointed out earlier in the example of the tablet case, a polymer component does not need to be broken into two pieces to cause it to be thrown away.

## 5. Conclusions

The goal of this work was to create a quantitative metric to evaluate the effect of damage and subsequent recovery on the tensile strength of polymers. The results showed that this effect varies, depending on the materials used and the manufacturing technique, where here the additive manufacturing process of fused filament fabrication was compared to injection molding. The ABS/SEBS blend showed nearly no loss of strength in the 3D printed form after being recovered from varying amounts of deformation, while the injection molded samples showed a decrease in average tensile strength. This showed a sensitivity to the manufacturing method of the samples for this polymer blend. The opposite was true for the PLA/SEBS blend, in that it showed no sensitivity to manufacturing method but rather a sensitivity to whether the sample was allowed to dwell under load during deformation. The PLA/SEBS blend showed a worse healing parameter when dwell time was included in the test schedule and a better performance when there was no dwell time. However, dwell time had a significant effect on the shape memory properties of the blends, as indicated by the improvement in the fixation ratio across the board for specimens that were held under load for five minutes. The PLA/SEBS performed the best in shape memory characterization but not for the resilience parameter, while the opposite was true for the ABS/SEBS blend, which did not perform well as a shape memory polymer but performed very well in terms of the healing parameter, particularly in 3D printed form. Though we evaluated processing the PLA/TPU blend, injection molded specimens that were allowed to dwell under load exhibited an increase in strength after being thermally recovered from deformation, a phenomenon most-likely related to crystallization of the material.

Shape memory polymer research must continue in earnest, so that we may quell the ever-increasing amount of polymer waste we generate as a civilization. We believe that evaluating materials using their resilience parameter could be very useful in developing polymer systems for making products with significantly longer lifespans, thanks to their ability to recover from damage.

## Figures and Tables

**Table 1:** Critical parameters and the metrics affected in this study.

Parameter	Mechanical Properties	Shape Memory Properties	Resilience Parameter
Dwell Time	X	X	X
Strain Before Recovery	X	X	X
Fabrication Method	X	X	X
Recovery Temperature		X	

**Table 2:** Extrusion parameters for the blends used in this study.

	PLA/SEBS	ABS/SEBS	PLA/TPU
Zone 1 (°C)	175	200	180
Zone 2 (°C)	180	215	185
Zone 3 (°C)	180	215	185
Zone 4 (°C)	180	205	185
Zone 5 (°C)	175	190	180
Zone 6 (°C)	175	190	175
Barrel Pressure (Bar)	90	90	90
Barrel Screw Speed (RPM)	16 *	12 *	14 *
Melt Pump Speed (RPM)	12	12	12

* Extruder varies the barrel screw speed continuously to maintain the pressure set point of 90 bar.

**Table 3:** FFF machine parameters for the blends used in this study.

	PLA/SEBS	ABS/SEBS	PLA/TPU
Nozzle Temperature (°C)	230	250	250
Bed Temperature (°C)	60	90	60
Nozzle Diameter (mm)	0.5	0.5	0.5
Infill Percentage (%)	100	100	100
Layer Height (mm)	0.2	0.2	0.2
Printing Speed (mm/min)	1800	1800	1800

**Table 4:** Injection molding temperatures for the blends used in this study.

	PLA/SEBS	ABS/SEBS	PLA/TPU
Injection Temperature (°C)	200	210	200
Mold Temperature (°C)	25	55	25
Preheat Time (min)	5	5	5

**Table 5:** DMA test parameters for the blends used in this study.

	PLA/SEBS	ABS/SEBS	PLA/TPU
Initial Temp (°C)	−40	−80	−80
Final Temp (°C)	110	150	100
Heating Rate (°C/min)	2	2	2
Frequency (Hz)	1	1	1

**Table 6:** Average values for the shape memory properties for the PLA/SEBS blend for 3D printed (3DP) and injection molded (IM) specimens.

Group	Amount of Initial Deformation											
	25%				50%				100%			
	*R* *_f_*	*σ*	*R* *_r_*	*σ*	*R* *_f_*	*σ*	*R* *_r_*	*σ*	*R* *_f_*	*σ*	*R* *_r_*	*σ*
3DP ^1^	81.6	0.2	100.9	0.1	83.5	0.2	100.4	0.2	87.3	0.5	100.2	0.2
3DP Dwell ^2^	88.9	2.5	101.2	0.2	88.4	3.0	100.6	0.05	90.1	0.1	100.3	0.01
IM ^1^	60.6	2.8	100.4	0.1	69.4	2.4	100.1	0.03	74.3	1.0	100.0	0.02
IM Dwell ^2^	81.7	2.6	100.4	0.1	85.6	1.0	100.1	0.03	86.6	0.4	100.0	0.01

^1^ Sample size *n* = 5. ^2^ Sample size *n* = 3.

**Table 7:** Shape memory properties for the ABS/SEBS blend for 3D printed (3DP) and injection molded (IM) specimens.

Group	Amount of Initial Deformation											
	25%				50%				100%			
	*R* *_f_*	*σ*	*R* *_r_*	*σ*	*R* *_f_*	*σ*	*R* *_r_*	*σ*	*R* *_f_*	*σ*	*R* *_r_*	*σ*
3DP ^1^	40.0	0.1	101.8	0.3	49.0	0.1	100.8	0.04	58.0	0.6	100.4	0.1
3DP Dwell ^2^	45.1	1.4	101.4	0.1	48.3	0.4	100.7	0.03	56.2	1.6	100.3	0.02
IM ^1^	38.0	0.8	101.8	0.4	48.6	1.6	100.8	0.1	60.7	1.0	100.3	0.03
IM Dwell ^2^	49.1	0.9	102.0	0.3	56.0	0.3	101.0	0.1	65.4	0.7	100.5	0.03

^1^ Sample size *n* = 5. ^2^ Sample size *n* = 3.

**Table 8:** Shape memory properties for the PLA/TPU blend for 3D printed (3DP) and injection molded (IM) specimens.

Group	Amount of Initial Deformation											
	25%				50%				100%			
	*R* *_f_*	*σ*	*R* *_r_*	*σ*	*R* *_f_*	*σ*	*R* *_r_*	*σ*	*R* *_f_*	*σ*	*R* *_r_*	*σ*
3DP ^1^	74.9	1.5	100.2	0.1	76.0	1.0	100	0.04	80.0	3.8	99.9	0.01
3DP Dwell ^2^	80.9	2.9	100.3	0.01	83.7	2.3	100	0.05	85.4	1.2	99.9	0.02
IM Dwell ^2^	86.7	1.9	99.9	0.03	88.7	0.3	100	0.01	87.9	0.4	99.6	0.1

^1^ Sample size *n* = 5. ^2^ Sample size *n* = 3.

**Table 9:** Determination of the resilience parameter based on linear fitting.

	Group	Line of Best Fit	R^2^ Value	Resillence Parameter (*m*/*y*)
PLA/SEBS	3D	*y* = −3.3897*x* + 25.308	0.983	−13.39
	3D Dwell	*y* = −3.7657*x* + 25.16	0.8216	−14.97
	I.M.	*y* = −2.1051*x* + 19.696	0.6311	−10.69
	I.M. Dwell	*y* = −3.2834*x* + 26.344	0.7055	−12.46
ABS/SEBS	3D	*y* = −0.0274*x* + 20.412	0.0097	−0.13
	3D Dwell	*y* = −0.0571*x* + 19.77	0.0023	−0.29
	I.M.	*y* = −2.1051*x* + 19.696	0.6311	−10.69
	I.M. Dwell	*y* = −1.4949*x* + 22.134	0.9339	−6.75
PLA/TPU	3D	*y* = −17.003*x* + 26.364	0.7648	−64.49
	3D Dwell	*y* = −2.0526*x* + 27.808	0.1168	−7.38
	I.M.	-	-	-
	I.M. Dwell	*y* = 11.841*x* + 33.192	0.9566	35.67

## Data Availability

Data sharing is not applicable to this article.

## Supplementary Materials

A video illustrating the shape memory properties of the PLA/SEBS blend can be seen at the following link: https://www.youtube.com/watch?v=vbe5mA5xY5k (accessed on 16 August 2023).
